# A Microplate Reader-Based System for Visualizing Transcriptional Activity During *in vivo* Microbial Interactions in Space and Time

**DOI:** 10.1038/s41598-017-00296-4

**Published:** 2017-03-21

**Authors:** Rosanna C. Hennessy, Peter Stougaard, Stefan Olsson

**Affiliations:** 1 Department of Plant and Environmental Sciences, University of Copenhagen, Frederiksberg C, 1871 Denmark; 2 State Key Laboratory of Ecological Pest Control for Fujian and Taiwan Crops, Fujian Agriculture and Forestry University, Fuzhou, 35002 China

## Abstract

Here, we report the development of a microplate reader-based system for visualizing gene expression dynamics in living bacterial cells in response to a fungus in space and real-time. A bacterium expressing the red fluorescent protein mCherry fused to the promoter region of a regulator gene *nunF* indicating activation of an antifungal secondary metabolite gene cluster was used as a reporter system. Time-lapse image recordings of the reporter red signal and a green signal from fluorescent metabolites combined with microbial growth measurements showed that *nunF*-regulated gene transcription is switched on when the bacterium enters the deceleration growth phase and upon physical encounter with fungal hyphae. This novel technique enables real-time live imaging of samples by time-series multi-channel automatic recordings using a microplate reader as both an incubator and image recorder of general use to researchers. The technique can aid in deciding when to destructively sample for other methods e.g. transcriptomics and mass spectrometry imaging to study gene expression and metabolites exchanged during the interaction.

## Introduction

Microbial interactions are found in numerous biological processes ranging from mixed infections in humans, animals and plants, to the development of fermented foods or the treatment of waste water. Thus, understanding how microbes coexist and interact in such communities is essential for fighting polymicrobial infections and for the development of robust, mixed microbial cultures for applications within biotechnology. In order to achieve this, a technology toolbox is essential. Current technologies available for studying microbial interactions include gene expression techniques (qPCR^1^, microarrays^2, 3^, and RNA sequencing^4, 5^), the identification of secondary metabolites by imaging mass spectrometry (IMS)^6–8^ methods and tools for gene deletion, replacement or expression control such as CRISPR-Cas9 technology^9^. Previously, we demonstrated that a combination of genomics, molecular genetics and microbiology coupled with IMS analysis could be used to unravel the mode of action underlying the antagonism of *Pseudomonas fluorescens* In5 against diverse phytopathogens^10^. More recently, we have also used transcriptomics to study gene expression in this biocontrol bacterium during phytopathogen interactions (manuscript in preparation). However, a disadvantage of transcriptomics and IMS analysis is the destructive nature of the technologies which are difficult to adapt for *in vivo* measurements to provide space and time information. Furthermore, current technologies can be time-consuming and expensive requiring sampling at numerous specific time points in order to study the dynamics of an interaction between microorganisms. Therefore, to investigate in more detail the interaction of our model organism *P. fluorescens* strain In5 with a phytopathogen, we have adapted a standard microplate reader as a tool for image-based real-time gene expression analysis of living cells.

*P. fluorescens* strain In5 has a high potential for secondary metabolite production and recently, the two non-ribosomal peptides (NRPs) nunamycin and nunapeptin have been shown to underpin the antimicrobial activity of this isolate against diverse phytopathogens including *Fusarium graminearum* ^10, 11^. Functional characterization of this strain during interactions with pathogens relies on assays where the bacterium is grown in co-culture with a pathogen on solid media. Many secondary metabolite gene clusters are silent under standard laboratory conditions and in particular fungi often produce increased amounts of secondary metabolites when cultivated on agar-based surfaces compared to in liquid media^12^. We have observed that culture conditions also play an important role in secondary metabolite production for strain In5. To date, only nunapeptin production has been detected in liquid media, whereas both peptides are produced on agar-based media. In addition to nunamycin and nunapeptin, strain In5 also produces a green fluorescent compound, most likely the iron chelating siderophore pyoverdine, which is known to be synthesized by several pseudomonads^13^. Thus a key challenge in the analysis of such interactions is the non-destructive measurement of target gene expression in space and in real-time using living cells in co-culture on solid surfaces.

Reporter strains that conditionally express fluorescent proteins (e.g. mCherry or GFP) are useful tools for gene expression profiling in living cells. Such strains can be monitored in microtiter plates grown in liquid medium and used to indicate for example the production of a compound whereby increased fluorescence from the fluorophore fused to a selected promoter or gene will signal the upregulation of biosynthetic genes. The fluorescent protein signal dynamics can then be measured in relation to biomass growth as an indicator of secondary metabolite production per cell over time.

Here, we describe a microplate reader-based imaging system for studying living microbial interactions and document the application of this technique combined with a reporter strain as a tool for monitoring gene expression in both space and real-time. For this we used living cells of *P. fluorescens* strain In5 growing on an agar surface confronting an approaching growing hyphal front of the phytopathogenic fungus, *F. graminearum*. Moreover, we discuss the advantages of this technology and how it can be used in many other applications where microorganisms are co-cultivated or form mixed species consortia, *e.g*. microorganisms associated with plant roots or leaves, wound infections or biofilms.

## Results

In strain In5, the *nunF* gene encoding a putative LuxR-type regulator is required for both nunamycin and nunapeptin synthesis. In addition, site-directed mutagenesis of the *nunF* gene in strain In5 renders the mutant incapable of suppressing the growth of phytopathogens. In this study, we specifically wanted to examine the rate of antifungal peptide production on an agar-based surface in response to a fungal colony gradually growing into closer contact with the reporter bacterial colony. This was achieved by placing the promoter region located upstream of the start codon of our gene of interest *nunF* in front of an mCherry fluorescent protein-encoding gene located on a medium-copy plasmid. A reporter strain was constructed by inserting the assembled plasmid into our model strain In5. A bacterial-fungal interaction could then be established on an agar-filled Nunc OmniTray to enable the recording of the microbial interaction in space and in real-time resembling a setting somewhat similar to time-lapse photography.

**Figure 1:**
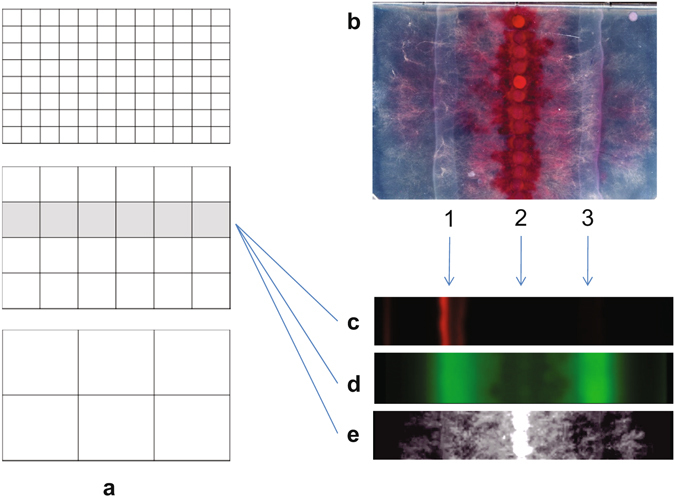
Recording images of plates using a plate reader. (**a**) The plate area with the interacting fungus can be defined as containing 96, 24 or 6 “wells” with zero thickness walls that can each be scanned (30 × 30 readings). (**b**) Scan of a Nunc OmniTray plate (120 × 80 mm) (Thermo Fisher Scientific, Waltham, MA) using a standard flatbed optical document scanner containing the fungus *F. graminearum* inoculated, with a row of agar plugs cut from a colony, at position 2. *P. fluorescens* In5 containing a plasmid with *nunF*-promoter-mCherry construct was inoculated as a streak at position 1 and the bacterium containing a plasmid with the same construct but lacking the promoter was inoculated as another streak at position 3. (**c–e**) Images constructed from scans by the BMG LABTECH microplate reader (BMG LABTECH, Offenburg, Germany) in different channels of the same row of 6 “wells”. (**c**) Red fluorescence (Excitation filter 584 nm Emission filter 620–10 nm) recording the mCherry signal. (**d**) Green fluorescence (Excitation filter 485-12 Emission filter 520) recording the bacterial fluorescent siderophore pyoverdine and also a fungal fluorescent compound. (**e**) Absorbance 660 nm measuring optical density (OD) used to measure biomass (660 nm is not absorbed by the red fungal pigment).

Firstly, the fungus *F. graminearum* was inoculated as a line of 6 mm agar plugs with fungal mycelia down the middle of the Nunc OmniTray in position 2 (Fig. 1b). The reporter strain In5 harbouring the plasmid carrying the *nunF* gene promoter region fused to mCherry was streaked 3 cm away from the fungus at position 1 (Fig. 1b) and the control strain In5 containing a plasmid carrying a promoter-less mCherry fusion was streaked at position 3 (Fig. 1b). Some standard microplate readers including the FLUO-star Omega model by BMG LABTECH have an integrated well scanning function with the possibility to define non-standard plates including those with both square and circular wells. As single-well plates with a working volume of 35 mls (i.e. Nunc OmniTray) are of the same format as a 96-well microtiter plate, we selected the OmniTray plate for imaging of, in this case, microbial interactions on agar. Using the BMG Omega software (i.e. program required for running the plate reader) plates were defined and 3 plate definitions were set up for 6, 24 or 96 square wells with no wall thickness between so-called “wells” (Fig. 1a, see also Supplementary File S4). When test protocols are set to use the scanning mode with 30 × 30 readings per “well”, it is possible to image the entire OmniTray at low- (90 × 60), medium (180 × 120) and high resolution (360 × 240). A low resolution scan of an entire OmniTray is completed within less than 15 minutes whereas scanning at high resolution can take up to 4 hours. It is relatively simple to instruct the microplate reader to scan a defined area of “wells” or alternatively a strip of “wells” (Fig. 1a middle section).

The standard BMG Omega Mars software (BMG LABTECH) is not adapted for imaging scanning outputs. However, test run readings can be exported in a matrix format to enable an ASCII text file (TXT) (one clean table of ASCII values for each measured pixel point of the same format, width and breadth as the final image) to be prepared in MS Excel and then saved as a text file. This file can subsequently be imported into the free image analysis software ImageJ (http://imagej.nih.gov/ij/) as a text image resulting in a 32-bit floating point image in ImageJ (Fig. 1c,e). In ImageJ, background blanks can be subtracted and the signal intensity in regions of interest of any shape can be recorded coupled with intensity along a line profile defined by either a line or rectangle. A notable feature of ImageJ is the option to use image mathematics as a tool for subtracting an image from images taken at later time points for time-series analysis. Consequently, the technique described above can lend itself to multiple applications such as high-throughput screening of colonies spread on plates to monitor enzyme activity over time or colour changes indicating active enzymes.

In contrast to the above, the application of time-series recordings for animations is slightly more complicated. The BMG Omega software has the capacity to run scripts which allows for the customisation of test runs using different test protocols. Using both the script wizard combined with the script tool in the BMG Omega software, a simple script was written that uses a “fluorescent test protocol” measuring two different fluorescent signals followed by an “absorbance test protocol” for recording relative biomass as OD^14, 15^ (see Supplementary File S1). A new version of the MARS software (Omega V5.11) was supplied by BMG LABTECH to enable a set of test runs to be exported as tab limited tables of “well” data to separate text files. A simple program was then written that can batch convert MARS formatted text files in a folder to text image format (see Supplementary File S2). These files can subsequently be imported into ImageJ to build a time-series image stack. A step-by-step guide for “Defining plates in the BMG Omega software”, “Single time recording of an image of a Nunc OmniTray using a plate reader and test protocol and transfer of data image to ImageJ” and “Time-series recording of images using a plate reader using several test protocols and transfer of data to ImageJ” can all be viewed in the Supplementary material (see Supplementary File S4).

**Figure 2:**
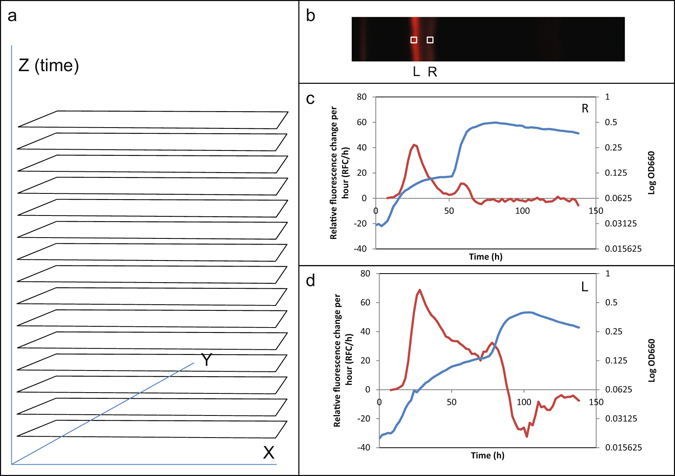
Constructing a stack of images with a time dimension and using this for analysis. (**a**) Images of the area of interest (as in Fig. 1c) are stacked along a time axis Z. (**b**) Two regions of interest were defined, one at the right side (R) of the bacterial reporter strain streak that met the growing fungus first and one at the left (L). (**c**) Plot of log OD (blue and right scale) showing an initial exponential growth of the bacterium and the arrival of the fungal colony front, made up of tip growing fungal cell filaments (hyphae), at approximately 50 h. The red curve (left scale) shows fluorescence change (mCherry protein synthesis rate) per hour of the reporter gene product, indicating changes in expression rate of all genes regulated by the *nunF* promoter. The mCherry synthesis rate has a peak in the deceleration phase of the growth and a second peak is seen at the arrival of the hyphal front. (**d**) Same as in (**c**) but since the arrival of the hyphal front is later the second peak is later and also here it coincides with the arrival of the hyphal front, at approximately 80 h.

The resulting image stack can be turned into an animation presentation (see Supplementary video) or analysed using the ImageJ program. A useful feature of ImageJ is the option to plot the average intensity for a region of interest through the whole stack using the “plot z-axis profile” (Fig. 2b–d). Using this method, a second peak in the reporter gene expression, upon contact of the fungus with the bacterial reporter streak, was detected first in the area closest to the fungus (Fig. 2c) and secondly at a slightly later time point in an area further away from the fungus (Fig. 2d). By selecting a region of interest just outside the bacterial streak on the side facing the fungus, it could be confirmed that the second increase in OD shown in Fig. 2c,d was due to the arrival of the fungal front (see Supplementary Figure S5). This demonstrated that the NunF-regulated gene transcription is switched on both when the bacterium enters the deceleration growth phase and when the bacterium physically encounters fungal hyphae. The peak in biosynthesis of the mCherry reporter is higher on the left side than on the right side of the streak facing the fungus. This could be the result of the fungus producing translation-inhibiting mycotoxins such as trichotecenes and zearaleone^16, 17^ that might negatively affect the formation of the mCherry and more affect the side of the bacterial streak facing the fungus. A weak signal of a fungal-produced fluorescent compound that might be a zearalenone^18^ was also seen accompanying the expanding fungal mycelium growth signal (OD) (see Supplementary video).

Consequently, using a standard plate reader, a reporter strain could serve as a tool to indicate the upregulation of the secondary metabolite gene clusters encoding for nunamycin and nunapeptin in response to the presence of the fungal pathogen *F. graminearum*.

## Discussion

Previously, using a combination of functional genomics and matrix-assisted laser desorption IMS analysis we described the identification of an antifungal genomic island encoding the two secondary metabolites nunamycin and nunapeptin and showed that both metabolites play a protective role against plant pathogens^10^. Here, we examined the application of the above described method for investigating the expression of a regulator of secondary metabolite synthesis in strain In5 during co-culture with the fungal pathogen *F. graminearum*. We used this technique to examine expression of a target gene during a live microbe-microbe interaction. While this technique was tested on a bacterial-fungal interaction, it can be used to analyze microbial interactions of choice provided it can be evaluated on a solid agar surface using optical density and/or fluorescence measurements. Essentially, this method allows for the quantitative multi-channel optical imaging of surfaces which can be extended to multiple applications. An obvious application of this method would be the screening of mutant bacterial colonies (*e.g.* strain collections) spread across agar plates such as the OmniTray plates particularly if it can be combined with a BMG Stacker (or similar device) for automated scanning of numerous plates. Another function that may interest many labs would be to ability to record the development of clearing zones over time in enzyme-based screens or antibiotic screening programs. For both fungal and bacterial colonies there are also marked differences in gene expression of the various areas of the colonies during development that could be monitored over time using this technique and evaluated depending on the study context. Providing a lab has access to a microplate reader, the method is cost-effective as only the appropriate plate is required for use of the method. As previously discussed, mass spectrometry imaging enables the spacial localization of compounds during microbial interactions across surfaces^19^. One of the main disadvantages of this technology is the destructive nature of the sampling method which requires sacrificing the growth system. In addition, some compounds of interest may not ionize and therefore a mass spectrum will not be obtained. Linking chemotypes at the transcriptome level using for example RNA-sequencing provides a genome-wide view of genes and their expression patterns during interactions. However, disadvantages include choosing the correct time point for analysis in addition to technical limitations such as obtaining sufficient RNA yields at high purity and potential impacts of sample treatment or degradation on transcript quantification. As the technology continues to develop the cost is decreasing however it is still a limitation for detailed time-studies under different conditions. While the method here developed also has limitations such as maturation time of fluorescent protein markers and requires known genes and metabolites for analysis, the proposed imaging technique will make it possible to closely monitor diverse interactions in combination with analyzing the expression of specific reporter genes to determine key time points for subsequent IMS analysis of the same plate in addition to enabling the determination of suitable time-points for transcriptomic analysis.

## Conclusion

In conclusion, the method here described enables the monitoring of changes in gene expression patterns over time allowing for the accurate determination of key-time points during an interaction that can be subsequently selected for mass spectrometry imaging analysis in order to determine the presence and spatial distribution of molecules and/or for global transcriptomics profiling. The limitation of this method is the requirement for fluorescent molecules and the time required for their maturation. The main advantage is the sampling method which compared to IMS analysis or omics-based technologies is non-destructive. On its own, this technology will enable the monitoring of gene expression in living cells in space and in time, and together with existing technologies (transcriptomics, IMS) will facilitate in-depth studies linking genotype, phenotype, and chemotype. Importantly, this method expands the technology toolbox currently available for studying microbial interactions.

## Additional Method Information

### Strains and Culture Conditions

*P. fluorescens* In5^10^ was routinely cultured on Luria-Bertani (LB) agar supplemented with 50 µg ml^−1^ kanamycin. Fungal strain *F. graminearum* PH-1^20^ was routinely sub-cultured on Defined Fusarium Medium (DFM)^20^.

### Construction of a mCherry-based reporter of *nunF* gene expression

A 467 bp fragment containing the predicted promoter region upstream of the *nunF* start codon was amplified using LongAmp™ *Taq* DNA polymerase (NEB) from *P. fluorescens* In5 genomic DNA using the forward (5′-CACAGGAGGCCGCCTAGGCCGCGGCCGCGCGAATTCGCCGACCGTTGGTCGGCTTTGTC-3′) and reverse (5′-GCTTGCATGCCTGCAGGTCGACTCTAGAGGATCCAAGCCTGCATACCAAAATCGCTG-3′) primers to yield a 468 bp product. The PCR product was then cloned using Gibson Assembly (NEB, BioNordika, Herlev, Denmark) into *Bam*H1-*Eco*R1 digested pSEVA237R^21^ upstream of an mCherry- expressing cargo. Fusion of the amplicon and plasmid was confirmed by restriction digest of plasmid DNA followed by Sanger sequencing (GATC-Biotech, Konstanz, Germany) to confirm integrity of the DNA sequence. The resultant construct was then transformed into strain In5 by electroporation as previously described^10^.

## Dual-culture assay

Nunc OmniTray (Fisher Scientific, Roskilde, Denmark) sterile plates were filled with 35 ml of tenth strength DFM^20^ and ten 6 mm diameter plugs of the ascomycete fungus *Fusarium graminearum* PH-1 were placed in a line across the middle of the plate and incubated 48 hours at 28 °C in the plate reader. The reporter strain In5 harboring either the empty vector control (*mCherry*) or the *nunF* promoter region fused to mCherry (*mCherry::PnunF*) were grown in 10 ml Luria-Bertani broth supplemented with 50 µg ml^−1^ kanamycin overnight at 28 °C and subsequently streaked in a line 3 cm away from, and parallel to the fungal plugs using a sterile inoculation loop.

## Electronic supplementary material

Supplementary Information

Supplementary Video
