# Supplementary material for: A Microplate Reader-Based System for Visualizing Transcriptional Activity During in vivo Microbial Interactions in Space and Time
Source: Sci Rep. 2017 Mar 21;7:281. doi: 10.1038/s41598-017-00296-4 (PMC5412646; doi:10.1038/s41598-017-00296-4)
Supplement: Supplementary file 1 — Supplementary Information [file 41598_2017_296_MOESM1_ESM.doc]

**Supplementary information for:**

**A Microplate Reader-Based System for Visualizing Transcriptional Activity During *in vivo* Microbial Interactions in Space and Time.**

**Hennessy RC^1^, Stougaard P^1^, Olsson S^1*^**

^1^ Section for Microbial Ecology and Biotechnology, Department of Plant and Environmental Sciences, University of Copenhagen. Thorvaldsensvej 40, DK-1871 Frederiksberg C, Denmark

*Corresponding author e-mail: sto@plen.ku.dk or [stefan@olssonstefan.com](mailto:stefan@olssonstefan.com)

**Content (page No)**

1. Supplementary File S1. Script for running fluorescence and absorbance image scanning protocols at 2h time intervals.
2. Supplementary File S2. MS Windows batch converter program from MARS text file export to text images (zip file containing an executable file and the instructions for use of the executable file).
3. Supplementary File S3. Macro script for import to ImageJ of a series of text images in the same folder to make an image stack.
4. Supplementary File S4. Step-by-step instructions for Defining plates, Single time recording of an image using one test protocol and Time series recording of images using a combination of test protocols.
5. Supplementary Figure S5. The arrival of the hyphal front to the bacterial streak.
6. Supplementary video legends

**Supplementary File S1. Script for running fluorescence and absorbance image scanning protocols at 2h time intervals.** The script was generated by Script Wizard (Omega v5.10 R2) and further edited as indicated in Supplementary file S4.

**
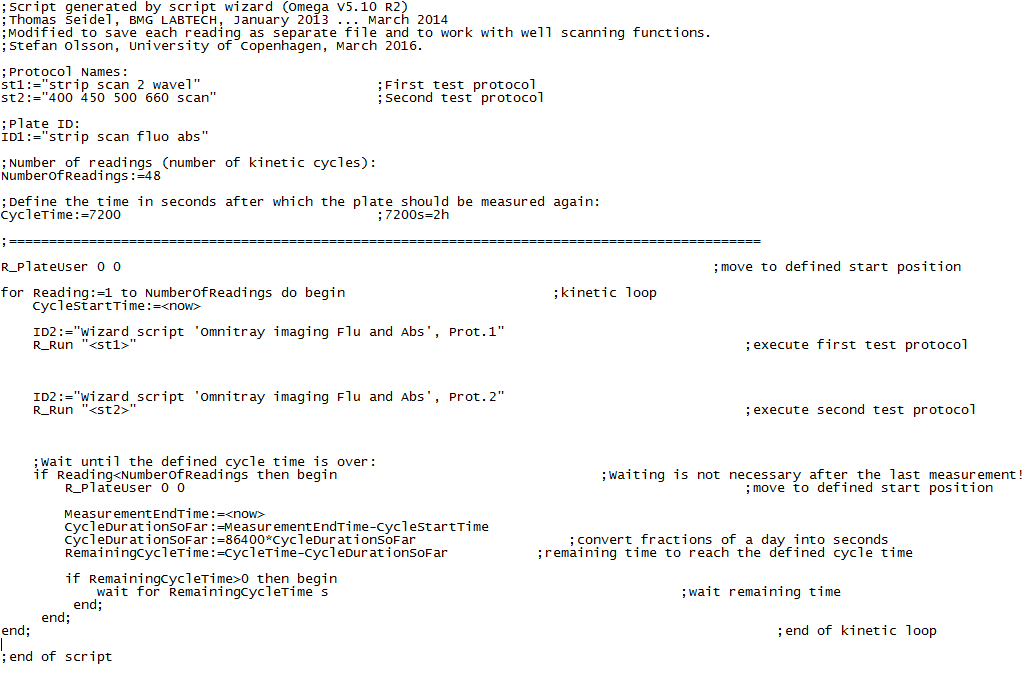
**

**Supplementary File S2. MS Windows batch converter program from MARS text file export to text images (zip file containing an executable file and the instructions for use of the executable file).**

1. Mark a set of test run data for export into the MARS “Manage Test Run” view. Right click and select Export “Multiple ASCII files…”. Select a folder to store the text files in. By using the “Bulk Rename” function and insert “zeroes” see to that the file index numbers have the same number of digits (important if file index numbers are larger than 100).

2. Double-click the converter program MofM.exe that can be placed in any folder and does not require installation. In the dialogue, enter the full path name (note: use double backslash \\ in the path name) of the folder containing the text files saved in step 1. Give a prefix for the converted files. Once the converter program has run, a folder containing the converted files is created. These converted files can then be used for “text image series import to stack” using the ImageJ Macro provided in Supplementary File S3.

**Supplementary File S3. Macro script for import to ImageJ of a series of text images in the same folder to make an image stack.**


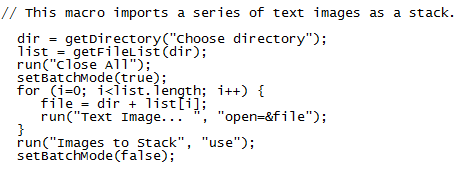


**Supplementary File S4. Step-by-step instructions for Defining plates, Single time recording of an image using one test protocol and Time series recording of images using a combination of test protocols.**

**A. Defining plates in the BMG Omega software (v.5.10 R2)**

Click the “Settings” tab and select “Manage Microplates” for a dialogue window appear.

To scan a Nunc OmniTray with 6 X 4 = 24 square “wells” select the “XY Dimensions” tab and enter the following settings: Name= whatever name you choose for the microplate definition, Plate format = 24, Length = 127.76 mm, Width =85.47 mm, Corner well X=12.68 mm, Corner well Y=12.78, Well shape=square, Well width= 20.62 mm. These settings are such that the “wells” have no “walls” and the whole surface will be scanned resulting in 24 tiled images making one large image if combined.

**B. Single time recording of an image of a Nunc OmniTray using a plate reader using and test protocol and transfer of data to ImageJ**

1. Set up a “well scan” protocol. Under the Basic Parameters tab choose one of the plate definitions defined in A as the Microplate. (Manage Protocol in BMG Omega software (v.5.10 R2)).

2. Set the scanning resolution (we always used 30 x 30 for the wells regardless of well size) by defining the Well scan settings. For the 24 “well” plates we thus used Scan matrix: 30 x 30 and Scan width 20 mm.

3. Then set the appropriate wavelength parameters for the type of measurements to be executed (e.g. fluorescence or absorbance). Note that for fluorescence measurements it is necessary to perform a pilot scan to determine the appropriate gain settings to use.

4. On the “Layout tab”; select the area to be scanned as a rectangle of scanned wells.

5. On the “Concentrations / Volumes / Shaking tab”; check that the protocol does not include shaking.

6. Scan the plate using the new protocol.

7. Open the test run results using the BMG Omega Mars program.

8. On the “Home” tab click” Excel Report” in the heading and tick “Microplate View” and “Export detailed well scan data”. Untick all other boxes except maybe the “Add test run information” since this could follow to the Excel file if needed. Hit the “Export Report to Excel” button.

9. In MS Excel trim the table so that it only contains a table with measured values and with the dimensions of the whole scanned plate (right number of columns and rows).

10. Save the MS Excel sheet with the table as an Ascii file (Text (MS-DOS).

11. Open the Ascii fil as an “Ascii image file” from the ImageJ program and perform image analysis.

**C. Time series recording of images using a plate reader using a combination of several test protocols and transfer of data to ImageJ.**

1. Set up test protocols using one of the plate definitions defined in A.

2. All the test protocols should have the same resolution and measure the same rectangular area of “wells”.

3. Select appropriate wavelength settings for what needs to be measured by the different protocols.

4. See to that the protocols does not include shaking

5. Under the “Microplate” tab, open Wizard to make a multiple protocol script by hitting the “New Button” button and select “Create a new script using the wizard”. Choose the appropriate protocols (i.e. protocols to measure fluorescence and/or absorbance). Select the number of cycles required (i.e. time). Then hit “Check timing” and set a cycle time longer than the minimum cycle time. Untick the “Shake during idle time” and then “Combine data from different protocols” box. Click OK.

6. Right click the new script icon and select “Export to script file” and save the script file.

7. Press Ctrl+Alt+S to enter Script Mode. Load the script saved under 6.

Edit the “kinetic loop” so that it only contains the ID2 lines running the protocols and save the script.

If a script button is wanted hit the “Add to button” button.

8. Scan the plate by hitting the “Start” button. And wait until all runs are finished.

9. For each measured protocol do the following: Select the set of stored test runs using the BMG Omega Mars program “Open function” (use the program version V3.20 R2 that includes the new “Export multiple Ascii files” function). Sort the test runs so that it is easy to select all test runs with the same protocol. Mark the set of test runs and right-click. Select “Export multiple ASCII files”. Check settings by clicking the “Settings” button: Untick boxes in the “File content options”. In the “File Name and Locations options” do the following. A. Select folder to store the set of output text files (there will be one file for each reading time). B. File name: use the <automatic filename creation> option. C. Select “File Extension”=TXT “Separator”=TAB and “If file exist”=”rename old file adding date/time”. In “Auto mode and Manage Test Runs File Export options” only tick the “Export microplate view” option. Then press the “OK” button.

10. Eventually use the “Bulk Rename Here” function in MS Windows Explorer. Mark the files to rename. Right click and choose “Bulk Rename Here” and insert “zeroes” so that file index numbers have the same number of digits (important if file index numbers are larger than 100).

11. Double-click the converter program MofM.exe (in supplementary file S2) that can be placed in any folder and need no installation. In the dialogue enter full path name (note: use double backslash \\ in the path name) of the folder containing the text files saved in 6 (and eventually renamed in 7). Give a prefix for the converted files. The program is now run and a subfolder containing the converted files that can be used for text image series import to stack using the ImageJ Macro provided in the supplementary file S3 is created.

12. Download the ImageJ macro for ASCII image import to image stack (<http://rsbweb.nih.gov/ij/macros/ImportTextImageSequence.txt>) and install it in ImageJ. Alternatively edit a macro in ImageJ to look like the one in Supplementary file S3, store it and activate it.

13. In ImageJ use the macro in 12 to Import all the images in the folder created in 11 to make an image stack.

14. In ImageJ check that the image stack has the images in the right order with the first recorded image at the top (first image). If not reverse the stack using the Reverse function in ImageJ found under Image>Stacks>Tools.

15. Now ImageJ can be used to analyze the image stack. Please observe that the image stack is a set of 32 bit floating point images that needs conversion to other image formats if to be presented in more “normal” image handling programs.

Limitations: Only one measurement (wavelength setting) can be used for each protocol. On the other hand several protocols can be run after each other in the script that then will be stored as separate files that can be combined to one image stack for each type of measurement.

**Supplementary Figure S5. The arrival of the hyphal front to the bacterial streak.**


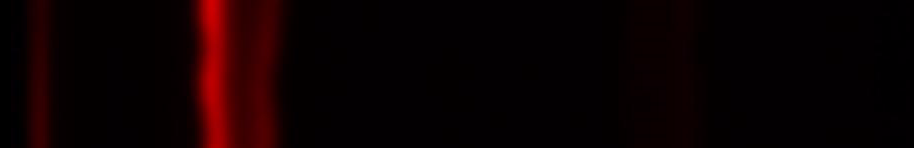


RO


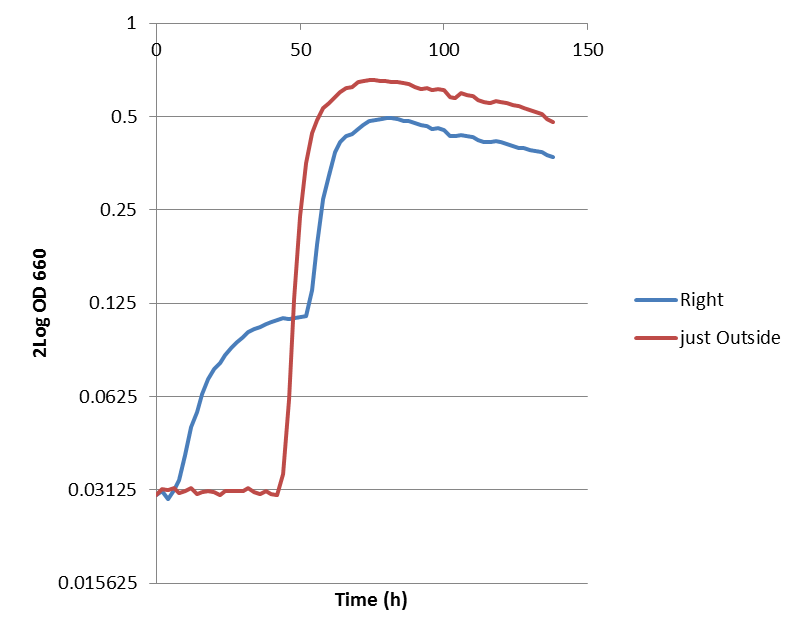


a

b

**Supplementary Figure S5.** The arrival of the fungal colony front to the bacterial streak as seen by optical density increase. (a) Image showing the areas of interest. R marks a region of interest at the right side of the bacterial reporter strain streak as in Figure 2 and O marks a region of interest just outside the bacterial streak facing the approaching fungal colony front. (b) The red curve shows the OD increase due to the arrival and growth of only the fungus in the O marked region. The blue curve shows the OD increase due to growth of the bacterium prior to the arrival of the hyphal front and then the OD Increase due to the arrival and growth of the fungus+bacterium after the arrival of the fungal colony front. Two observations of interest can be made. 1. The second increase in the blue curve is due to the arrival of the fungal colony front. 2. The maximum OD reached for bacterium + fungus in the R-marked area (blue curve) is less than for fungus alone (red curve).

**Supplementary Video legend**

**Time lapse video of the three strips shown in Figure 1c-e. The time between subsequent image frames are 2h.**

Top: mCherry signal. Left streak *Pseudomonas fluorescens* In5 with mCherry behind *nunF* promoter (plasmid) and right streak mCherry without promoter.

Middle: Fungal zearalenone(?) and bacterial pyoverdine signal.

Bottom: Optical density (OD) signal showing relative biomass.
